# Supplementary material for: SPEECHLESS duplication in grasses expands potential for environmental regulation of stomatal development
Source: bioRxiv. 2025 Oct 21:2025.07.29.667563. Originally published 2025 Jul 30. Preprint. [Version 2] doi: 10.1101/2025.07.29.667563 (PMC12327695; doi:10.1101/2025.07.29.667563)
Supplement: Supplement 1 — Fig. S1 Full amino acid alignment of SPCH1 and SPCH2 proteins from selected grass species Fig. S2 Predicted paralog-specific transcription factor binding motifs in 5’ regulatory sequences Fig. S3 Row density and stomatal spacing data for additional genotypes and environments Fig. S4 Protein accumulation of BdSPCH paralogs extends along the developmental zone in a paralog-by-condition specific manner Fig. S5 SPCH reporter per cell intensity is insufficient to explain paralog-specific environmental response Fig. S6 O. sativa stomatal density response driven by alternative mechanisms than those used by B. distachyon Table S1 Primer sequences used for genotyping and expression analysis Table S2 Data sources for SPEECHLESS (SPCH) paralog sequences used in this paper [file media-1.pdf]

SPEECHLESS duplication in grasses expands potential for environmental regulation of stomatal development

Authors: Joel M. Erberich, Britney Bennett and Dominique C. Bergmann

Fig. S1 Full amino acid alignment of SPCH1 and SPCH2 proteins from selected grass species
Fig. S2 Predicted paralog-specific transcription factor binding motifs in 5' regulatory sequences
Fig. S3 Row density and stomatal spacing data for additional genotypes and environments
Fig. S4 Protein accumulation of BdSPCH paralogs extends along the developmental zone in a paralog-by-condition specific manner
Fig. S5 SPCH reporter per cell intensity is insufficient to explain paralog-specific environmental response
Fig. S6 *O. sativa* stomatal density response driven by alternative mechanisms than those used by B. *distachyon*

Table S1 Primer sequences used for genotyping and expression analysis
Table S2 Data sources for SPEECHLESS (SPCH) paralog sequences used in this paper

```
                         |         |         |         |         | 1-55
AtSPCH       ---------MQEIIPDFLEECEFVDTSLAGDDLFAILESLEGAGEISPTAASTPK
HvSPCH1      ----MGDALCDQLLVD--VDGEFQ---LPTDAEDLFS-ILETWEDCVNG------
TaSPCH1B     ----MGDALCDQLLVD--VDGEFQ---LPTDAEDLFS-ILETWEDCVHG------
TaSPCH1A     ----MGDALCDQLLVD--VDGEFQ---LPTDAEDLFS-ILETWEDCVNG------
TaSPCH1C     ----MGDALCDQLLVD--IDGEFQ---LPTDAEDLFS-ILETWDDCVNG------
BdSPCH1      ---MGDIALCGQLLLDNVVDGEFQ---LPSDAEDLFS-ILETWEDCVNGSA----
OsSPCH1 (N)  ----MGDALCDQLLLVDSDGGEFIPHHADADADDLFT-ILETWEGCANVVAGGAP
OsSPCH1 (K)  ----MGDALCDQLLLVDSDGGEFIPHHADADADDLFT-ILETWEDCANVVAGGAP
PvSPCH1K     ----MAEALCDQLFSD--VDGELMMMQHPSDADDLLGSILEAWEDCVTGGGGATP
PvSPCH1N     ----MAEALCDQLFSD--VDAGELMMHHASDADDLLG-ILEAWEDCVTGGGCTTP
PhSPCH1      ----MEEALCDQLFSD--VDG-DLMMHHPSDADDLLG-ILEAWEDCVTGGGSTPR
SiSPCH1      ----MAEALCDQLFSD--VDG--ELMHHPSDADDLFG-ILEAWEDCVTGGGSTPR
SvSPCH1      ----MAEALCDQLFSD--VDG--ELMHHPSDADDLFG-ILEAWEDCVTGGGSTPR
SbSPCH1      ----MAEALCDQLFSD--VDG-ELMRQHTSDTDDLLG-ILEAWEDCVTGGGATTP
ZmSPCH1      ----MAEALCDQLFSD--IDG-DLMRQHTSDTDDLLG-ILESWEDCVSGGVATTP
SbSPCH2      ----MADGLCELLVDD---HCELRHRLSG--AEDLFS-IIGTTWKEGTNRAGGDG
ZmSPCH2b     ---MEDGGMCELVIGD---HRNLRHRLSG--AEDLFS-IVGTWEERTNGASGGGG
ZmSPCH2a     ----MGDGQCELVVDN---HCQLRHRLDLSGAEDLFS-VIGTWEERTDDGGAGDG
PvSPCH2K     ---MGDGAMCELLVDD---HSELR--HLS-GAEDLFS-ILETWEECVNGAGGGGG
PvSPCH2N     ---MGDGAMCELLVDD---HSELR--HQSSGAEDLFS-ILETWEECMAGSS----
PhSPCH2      ---MGDGAMCELLVDD---HSELR--HVS-GAEDLFS-ILETWEECMNGGGGGGG
SiSPCH2      ----MADGLCELMVDG---HRDLR--HLS-GAEDLFS-ILETWEECMNGGGGGGG
SvSPCH2      ----MADGLCELMVDG---HRDLR--HLS-GAEDLFS-ILETWEECMNGGGGGGG
OsSPCH2 (N)  MADGGGGGLCELFSDD---HRDIR----HVADADLFR-ILETWEECINGGAGGGG
OsSPCH2 (K)  MADGGGGGLCELFSDD---HRDIR----HVADADLFR-ILETWEECINGGAGGGG
BdSPCH2      MAMGDDGGLCKLFAND---DCEFR-----HVSGDLFS-ILDTWEKCMNGAGEGGG

                 |         |         |         |         |    56-110
AtSPCH       DGTTSSKELVKDQDYENSSPKR--------------------KKQRLETRKEEDE
HvSPCH1      --AAAALSPTAGAGSGGGLLG------------------GSAPGNKGSSKRRARQ
TaSPCH1B     --AAAALSPTAGASSRGELMG------------------GSAPGNK-SSKRRARP
TaSPCH1A     --AAAALSPTAGASSRGGLLG------------------GSAPGNK-GSKRRARQ
TaSPCH1C     --AATALSPTAGASSRGGLLG------------------GSASGNK-GSKRRARQ
BdSPCH1      --ATTALSPTSGASSGALLAPP-----------------DSKLGSKRRMAPQDQE
OsSPCH1 (N)  ATTTTLGSPIAAAACISGVVGGQNHQQLPEPAAAKTVPATNNKRREEEVADRDGD
OsSPCH1 (K)  ATTTTLGSPIAAAACISGVVGGQNHQQLPEPAAAKTVPATNNKRREEEVADRDGD
PvSPCH1K     RSAEPEVPQATDSTARASPPKP----------------------AAGNVRRMGDR
PvSPCH1N     RGGD-EVPQATDSTARASPPKP----------------------AAGNVRRMGDR
PhSPCH1      ---GAKVPQATDAAVATSNPAP----------------------ASGNVRRLGDR
SiSPCH1      G-----AEVSRTANATTPKPPS----------------------AVGSIRRLHDR
SvSPCH1      G-----AEVSRTANATTPKPPS----------------------AVGSIRRLHDR
SbSPCH1      RG---ADVLHQSASDAAATPKA----------------------AAAKRRRQGCR
ZmSPCH1      CG---AEVPQTTAADGAAK----------------------------RRRQGCR
SbSPCH2      AGG-CSSGAMRAYSQSSPAGA-----------------VGARPSVNCRKRTGD--
ZmSPCH2b     GGGGSSAAAVRAYSQGCTAGT-----------------AGAKTAAGTNNSRRR--
ZmSPCH2a     GGGGSSAPAMRAYRQISCSAG-----------------AVAATRLTGKSRRRTGE
PvSPCH2K     SSAAAMPAYSQSSTGGSESAG-----------------AVEGARPTANSRRRAGD
PvSPCH2N     AHAAAMPACSQSSTGGSESAG-----------------AMAGARPTANNRRRAGD
PhSPCH2      GSAAAMPAYSQSSTGGSESAG-----------------AVAGARPTANGRRRAGD
SiSPCH2      GGSSEAMPAYSQSSTGGSDSG-----------------AVAGARPAANSRRRSRD
SvSPCH2      GGSSEAMPAYSQSSTGGSDSG-----------------AVAGARPAANSRRRSRD
OsSPCH2 (N)  GGVSLAGVADQGAAAASTGAGGGA--------------RTTTTTTAANGRRREGR
OsSPCH2 (K)  GGVSLAGVADQGAAAASTGAGGGA--------------RTTTTTTAANGRRREGR
BdSPCH2      GAPCSAAALSPSSVVVVDGAAENN--------------AVVGVRPKPGSRRREAA
```

```
                         |         |         |         |   bHLH 111-165
AtSPCH        EEEDGDGEAEEDNKQDGQQ-----------------------KMSHVTVERNRRK
HvSPCH1       GECDGISHEHKRQKCSPEE-----------------GGGAAPKTAHITVERNRRK
TaSPCH1B      DECDGIAQTQKRQKCSPEE-----------------GGGAAPKTAHITVERNRRK
TaSPCH1A      DECDGIAQAQKRQKCSPEE-----------------GGGAAPKTAHITVERNRRK
TaSPCH1C      DDCDGTAQTHKRQKCSLEE-----------------GGGAAPKTAHITVERNRRK
BdSPCH1       DNDDTAAQAQKRRKCSP----------------------EAPKTAHITVERNRRK
OsSPCH1 (N)   GDDDDGSPQKRRKCCSPESS------------TTDVAAATTPKTAHIAVERNRRK
OsSPCH1 (K)   GDDDDGSPQKRHKCCSPESS------------TTDVAAATTPKTAHIAVERNRRK
PvSPCH1K      DDDATVPEAPKRQRCSPAMSS------SEAAATSDDGAANNNKTSHITVERNRRK
PvSPCH1N      DDATVPAAPPKRRRCSPAVSS------SEAAATSDDGAASN-KTSHITVERNRRK
PhSPCH1       ED-ATMRAAPKRRRCSPAVSS------SDAAATSDDGAANN-NTSHITVERNRRK
SiSPCH1       DQGDATVPAPKRQRCSPAVSS------EAAAATSEDGAANN-KTSHITVERNRRK
SvSPCH1       DQGDATVPAPKRQRCSPAVSS------EAAAATSEDGAANN-KTSHITVERNRRK
SbSPCH1       EEDGTAVPAPKRQRCSPVSS--------DAAAASEDGAANK--TSHITVERNRRK
ZmSPCH1       DVD-AVPAAPKRQKCSPVS-----------SAASEDGAVNK--TSHITVERNRRK
SbSPCH2       --EEKGSGGGAPVQKK--------HKGSAVTDDAAADEGEAKMSHITVERNRRK
ZmSPCH2b      --TGDEEKGGSAPAQKK--------HKGSSAVSD---DEGAAKMSHITVERNRRK
ZmSPCH2a      EEEEKGSGGSAPGPAHK--------KHNKAGSAVTDDDEGAPKISHVAVERNRRK
PvSPCH2K      EENGVGRGAPMRKKQKG--------SSTAAAQDAAADEG-AAKMSHIAVERNRRK
PvSPCH2N      EGNGVGRGAPAQKKQKG--------SSTAAAQDVAAADEGGAKMSHIAVERNRRK
PhSPCH2       EEKGVGRGAPAQKKQKG--------SSTAAAAQDAAADEGAVKMSHIAVERNRRK
SiSPCH2       EERGVGRGAPVPKKQKVS-------AAAAITAQDAAADEGAAKMSHIAVERNRRK
SvSPCH2       EERGVGRGAPVPKKQKVS-------AAAAITAQDAAADEGAAKMSHIAVERNRRK
OsSPCH2 (N)   DEEKGGGGGGGPPAQKKQKGSSSSSSSPAALAAAVGDGDGAAKMSHITVERNRRK
OsSPCH2 (K)   DEEKGGGGGGGPPAQKKQKGSSSSSSSPAALAAAVGDGDGAAKMSHITVERNRRK
BdSPCH2       DEEKGGAPGRK--------------KHKGSTVVDDGSDGAAKMSSHITVERNRRK

                  |         |         |         |         |   166-220
AtSPCH        QMNEHLTVLRSLMPCFYVKRGDQASIIGGVVEYISELQQVLQSLEAKKQRKTYAE
HvSPCH1       QMNEHLTVLRSLMPCFYVKRGDQASIIGGVVDYIKELQQVKQSLEAKKQRKAYTE
TaSPCH1B      QMNEHLTVLRSLMPCFYVKRGDQASIIGGVVDYIKELQQVKQSLEAKKQRKAYTE
TaSPCH1A      QMNEHLTVLRSLMPCFYVKRGDQASIIGGVVDYIKELQQVKQSLEAKKQRKAYTE
TaSPCH1C      QMNEHLTVLRSLMPCFYVKRGDQASIIGGVVDYIKELQQVKQSLEAKKQRKAYTE
BdSPCH1       QMNEHLAALRSLMPCFYVKRGDQASIIGGVVDYIKELQQVKQSLEAKKQRKAYTE
OsSPCH1 (N)   QMNENLAVLRSLMPCFYVKRGDQASIIGGVVDYIKELQQVLHSLEAKKQRKVYTD
OsSPCH1 (K)   QMNENLAVLRSLMPCFYVKRGDQASIIGGVVDYIKELQQVLHSLEAKKQRKVYTD
PvSPCH1K      QMNEHLAVLRSLMPCFYVKRGDQASIIGGVVDYIKELQQVLQSLEAKKQRKAYTD
PvSPCH1N      QMNEHLAVLRSLMPCFYVKRGDQASIIGGVVDYIKELQQVLQSLEAKKQRKAYTD
PhSPCH1       QMNEHLAVLRSLMPCFYVKRGDQASIIGGVVDYIKELQQVLQSLEAKKQRKAYTD
SiSPCH1       QMNEHLAVLRSLMPCFYVKRGDQASIIGGVVDYIKELQQVLQSLEAKKQRKAYTE
SvSPCH1       QMNQHLAVLRSLMPCFYVKRGDQASIIGGVVDYIKELQQVLQSLEAKKQRKAYTE
SbSPCH1       QMNEHLAVLRSLMPCFYVKRGDQASIIGGVVDYIKELQQVLQSLEAKKQRKAYTE
ZmSPCH1       QMNEHIAVLRSLMPCFYVKRGDQASIIGGVVDYIKELQQVLQSLEAKKQRKAYTE
SbSPCH2       QMNEHLTVLRSLMPCFYVKRGDQASIIGGVVDYIKELQQVLRSLETKKHRKAYAE
ZmSPCH2b      QMNEHLAVLRSLMPCFYVKRGDQASIIGGVVDYIKELQQVLRSLETKKHRKAYAE
ZmSPCH2a      QMNEHLTVLRSLMPCFYVKRGDQASIIGGVVDYIKELQQVLRSLEAKKHRKAYAE
PvSPCH2K      QMNEHLAVLRSLMPCFYIKRGDQASIIGGVVDYIKELQQVLRSLEAKKHRKAYAE
PvSPCH2N      QMNEHLAVLRSLMPCFYIKRGDQASIIGGVVDYIKELQQVLRSLEAKKHRKACAE
PhSPCH2       QMNEHLAVLRSLMPCFYIKRGDQASIIGGVVDYIKELQQVLRSLEAKKHRKAYAE
SiSPCH2       QMNEHLAVLRSLMPCFYVKRGDQASIIGGVVDYIKELQQVLRSLEAKKHRKAYAE
SvSPCH2       QMNEHLAVLRSLMPCFYVKRGDQASIIGGVVDYIKELQQVLRSLEAKKHRKAYAE
OsSPCH2 (N)   QMNEHLAVLRSLMPCFYVKRGDQASIIGGVVDYIKELQQVLRSLEAKKNRKAYAD
OsSPCH2 (K)   QMNEHLAVLRSLMPCFYVKRGDQASIIGGVVDYIKELQQVLRSLEAKKNRKAYAD
BdSPCH2       QMNENLAVLRTLMPCFYVKRGDQASVIGGVVDYIKELQQVLHSLEAKKHRKVYAV
```

```
                         |         |         |         |      221-275
AtSPCH        VLSPRVVPSP--------RPSPPVLSPRKPPLSPRINHHQIHHHLLLPPISPRTP
HvSPCH1       HVLSPRPPPS--------SYSPRLPLSPLHKSTPPL----SPLLRSTPPLSPRLA
TaSPCH1B      HVLSPRPPPT--------SYSPRLPLSPLHKSTPPL----SPLLRSTPPLSPRLA
TaSPCH1A      HVLSPRPPPS--------SYSPRLPLSPLHKSTPPL----SPLLRSTPPLSPRLA
TaSPCH1C      HVLSPRPPPS--------SYSPRLPLSPLHKSTPPL----SPLLRSTPPLSPRLA
BdSPCH1       QVLSPRPLP---------SPSPRLPLSPLLKSTPPL----SPRLATMS-------
OsSPCH1 (N)   QVLSPRPPAT--------VAASCCSPRPPQLSPRLP----PQLLKSTPPLSPRLA
OsSPCH1 (K)   QVLSPRPPAT--------VAASCCSPRPPPLSPRLP----PQLLKSTPPLSPRLT
PvSPCH1K      QVLSP----------------------RPPLPP---------LKSTPPISPRPS
PvSPCH1N      QVLSPRLPPA--------CCSPRPPLSPRPPLPP---------LKFTPPISPRPS
PhSPCH1       QVLSPRPPPA--------CCSPRPPLSPRPPLPP---------LKSTPPISPRPS
SiSPCH1       QVLSPRPPPP--------SCSPRPPLSPRPPLPP---------LKSTPPISPRPS
SvSPCH1       QVLSPRPPPP--------SCSPRPPLSPRPPLPP---------LKSTPPISPRPS
SbSPCH1       QVLSPR-PPA--------CCSPRPPLSPRPPLPP---------LKSTPPISPRPA
ZmSPCH1       QVLSPR-PPA--------CCSPRPPLSPRPHMLP---------LKSTPPISPRPA
SbSPCH2       QVLSPRPAGG--SVSAASPRPLAAVVKSTPPLSP----------RVAVPISPRTP
ZmSPCH2b      QVLSPRPLP---------------AVKSTPPLSP----------HVAVPMSPRTP
ZmSPCH2a      QVLSPRPSAGGVSTSVSAASPRHLAVKSVAPLSP----------RMAVPISPRTP
PvSPCH2K      QVLSPRPAVP-------AASPR-PLLKSTPPLSP----------RVAVPISPRTP
PvSPCH2N      QVLSPRPAMP-------AASPRPLLIKSTPPLSP----------RVAVPISPRTP
PhSPCH2       QVLSPRPAVS-------AASPR-PLLKSTPPLSP----------RVAVPISPRTP
SiSPCH2       QVLSPRTTVS-------AASPR-PLVKPTPPLSP----------RVAVPISPRTP
SvSPCH2       QVLSPRTTVS-------AASPR-PLVKPTPPLSP----------RVAVPISPRTP
OsSPCH2 (N)   QVLSPRPSPA----------AAALMVKPTPPISPRF----AAAAAAGVPISPRTP
OsSPCH2 (K)   QVLSPRPSPA----------AAALMVKPTPPISPRF----AAAAAAGVPISPRTP
BdSPCH2       EHALSPRPGP---------TPLSPRPLLKPPISP----------RPAVPISPPTP

                    |         |      PEST DOMAIN |         |   276-330
AtSPCH        QPTSPYRAIPPQLPLIPQPPLRSYSSLASCSSLGDPPPYSPASSSSSPSVSSNHE
HvSPCH1       VPISPARTPPTPGSPYKLRPLPP-------PISGSTYVSPAMTPTGYE---PGSS
TaSPCH1B      VPISPARTPPTPGSPYKLRPLPP-------PISGSTYVSPAMTPTGYE---PGSS
TaSPCH1A      VPISPARTPPTPGSPYKLRPLPP-------PISGSTYVSPAMTPTGYD---AASS
TaSPCH1C      VPISPARTPPTPGSPYKLRPLPP-------PISGSTYVSPAMTPTGYD---AASS
BdSPCH1       ----PCRTPPTPGSPYKLIRPLP-------LPPTMSSGSSAYVSPAMT---PTGC
OsSPCH1 (N)   VP-ISPRTPPTPGSPYRLLRLPP-------PPPPASGSNYASPAMTPT---HHET
OsSPCH1 (K)   VP-ISPRTPPTPGSPYRFLRLPP-------PPPPASGSNYASPAMTPT---HHET
PvSPCH1K      VPISPRTPPPGPGSLYKVRMQPP--LPLPLSPPGSAYASPARTPTREPSPASSSS
PvSPCH1N      FPISPRTPPPGPGSPYKVRRQPP--LPLPLSPPGSAYASPARTPTREPSPASSS-
PhSPCH1       VPISPRTPP-GPGSPYKVRRQPP--LPLPLSPPGSAYTSPARTPTREPSPASSS-
SiSPCH1       VLISPRTPP-TPGSPYKLRRQPPPPLPLPLSPPGSAYASPARTPTREPSPAPSY-
SvSPCH1       VLISPRTPP-TPGSPYKLRRQPPSPLPLPLSPPGSAYASPARTPTREPSPAPSY-
SbSPCH1       VPISPRTPPPAPSSPYKPRRQPI--SVPLPPPGSSAYASPAMTPTREP---AAAS
ZmSPCH1       VPISPRTPP-APSSPYKPCR--------LPPPGSSAYASPAMTTTREP---TAAT
SbSPCH2       TPGSPYKPAAAASGAAGTAGSCRLPYPPAAAAYMAAASPAITPTSSSSSYSLDQQ
ZmSPCH2b      TPGSPYKPASGAA--ATTTGSCRLPHRAAAAAAAYIGTPTTSSSSSSYSHDQ---
ZmSPCH2a      TPGSPYK-----AAASGVAGCCRLPLLPPYMLSSSAAAAAASYSHDQQQHYS---
PvSPCH2K      TPGSPYK--PSGGGGGGAASSRPPHPATAACYMMPSPAMTPTTSSSSSSYAHDQQ
PvSPCH2N      TPGSPYKQPSGGGGGSGAAAGSRPPHPAAAAYMMPSPAMTPTTSSSSSSYAHDQQ
PhSPCH2       TPGSPYK----PSGGGGGAGSSRLSHPAAAAYMIPSPAMTPTTSSSSSSYAHDHH
SiSPCH2       TPGSPYKPPPAGGAAAG------SRVPHPAAYMMPSPAMTPATSSSSSSYSHD--
SvSPCH2       TPGSPYKPPPAGGAAAG------SRVPHPAAYKMPSPAMTPATSSSSSSYSHD--
OsSPCH2 (N)   TPGSPYNKHAAAAATARPPHPAAATSSCSVAYSMSPAMTPTSSSSTTTTTTHELS
OsSPCH2 (K)   TPGSPYNKHAAAAATARPPHPAAATSSCSVAYSMSPAMTPTSSSSTTTTTTHELS
BdSPCH2       TPGSPYKPIQRLPHYISPATSSSSTISHAASYDVISPRP----------------
```

```
                         |          |          |          |        331-385
AtSPCH       S-------------------SVINELVANSKS-----------------------
HvSPCH1      --------------YLPSLDAIAAELSVYANRQALQLPPTDLL------------
TaSPCH1B     --------------YLPSLDAIAAELSVYANRQALQLPPTDPL------------
TaSPCH1A     --------------YLPSLDAIAAELSVYANRQALQLPPTDPL------------
TaSPCH1C     --------------YLPSLDAIAAELSVYANRQALQLPPTDPL------------
BdSPCH1      --------------HEPSLEAIAAELSVYAANRQATLLP----------------
OsSPCH1 (N)  --------------AAPSLDAIAAELSAYAS--RQALGGGLLL------------
OsSPCH1 (K)  --------------AAPSLDAIAAELSAYAS--RQALGGGLLL------------
PvSPCH1K     --------------YLPSLDKIAAELCAYAAGTSKQPQRPALLPAAAAGGGGGAA
PvSPCH1N     --------------YLPSLDKIAAELCAYAAGTSNKPQRPALLPATGGG---GVV
PhSPCH1      --------------YLPSLDKIAAELCAYAAGTNKQQQRQALLPAATSG--GAVV
SiSPCH1      ---------------LPSLDTIAAELCAYAARGTNKQQQAPVALPAAAG---GGG
SvSPCH1      ---------------LPSLDTIAAELCAYAARGTNKQQQAPVALPAAAG---GGG
SbSPCH1      --------------YLPSLDTVAADLCAYAATNK---QLQPALPAAAGG----VV
ZmSPCH1      --------------YLPSLDTIAADLCAYAANKN---KQLQALAAAAGG----DV
SbSPCH2      QQQQHYSMQTTTTTYLPTLDSLVTELAAQAACRPAAAGLN---------------
ZmSPCH2b     --------QRHYSTYLPTLDSLVTELAAQAAACSRPAASGG-------L-----T
ZmSPCH2a     ---------TQTTTYLPTLDSLVTELATQQAACRPAAAAAG-------------L
PvSPCH2K     ----QQLYPTTAQPYLPTLDSLVTELAARAAGG-RPAAAG--------------L
PvSPCH2N     ----QQQHYPTTQLYLPTLDSLVTELAARAAGG-RPAAAG--------------L
PhSPCH2      ----QQHYP-TTQPYLPTLDSLVTELAAQAAGG-RPAAAG--------------L
SiSPCH2      ----QQHYPATSQPYLPTLDSLVTELAAQAAGAGRPAAAG--------------L
SvSPCH2      ----QQHYPATSQPYLPTLDSLVTELAAQAAGAGRPAAAG--------------L
OsSPCH2 (N)  ----------PAPAFLPILDSLVTELAARGGASCRPLVIPSSAAAIAGI-----V
OsSPCH2 (K)  ----------PAPAFLPILDSLVTELAARGGASCRPLVIPSSAAAIAGI-----V
BdSPCH2      --------------YLPTLDSIITELAAQAAARAPGALGGGGGGAGLNL-----L

              SMF/ACT DOMAIN|          |          |          |   386-440
AtSPCH       ALADVEVKFSGANVLLKTVSHKI-PGQVMKIIAALED--LALEILQVNINTVDET
HvSPCH1      --PDVRVEFAGANLVLKTVSHRA-PGQAVKIIAALESRAPALEILHAKISTIDDT
TaSPCH1B     --PDVRVEFAGANLVLKTVSHRA-PGQVVKIIAALESRAPALEILHAKISTIDDT
TaSPCH1A     --PDVRVEFAGANLVLKTVSHRA-PGQAVKIIAALESRAPALEILHAKISTIDDT
TaSPCH1C     --PDVRVEFAGANLVLKTVSHRA-PGQAVKIIAALESRAPALEILHAKISTIDDT
BdSPCH1      ---DVRVEFRGANLVLKTVSPRA-PGQAVKIVAALEG--RALEILHAKISTVDDT
OsSPCH1 (N)  --PDVKVEFAGANLVLKTVSQRS-PGQAVKIIAALEG--RSLEILHAKISTVDDT
OsSPCH1 (K)  --PDVKVEFAGANLVLKTVSQRS-PGQAVKIIAALEG--RSLEILHAKISTVDDT
PvSPCH1K     LLPDVRVEFSGANLVVKTVSHRA-PGQAVKVIAALEG--RSLEILDAKISTVDDT
PvSPCH1N     LLPDVRVEFSGANLVVKTVSHRA-PGQAVKVIAALEG--RSLEILDAKISTVDDT
PhSPCH1      LLPDVRVEFSGANLVVKTVSHRE-PGQAVKVIAALEG--RSLEILDAKISTVDDT
SiSPCH1      LLPDVKVEFAGANLVVKTVSHRA-PGQAVKIIAALEG--RSLDILDAKISTVDDT
SvSPCH1      LLPDVKVEFAGANLVVKTVSHRA-PGQAVKIIAALEG--RSLDILDAKISTVDDT
SbSPCH1      VLPDVKVEFSGANLVVKTVSHRA-PGQTVKVIAALEG--RSLEILDAKINTVNDT
ZmSPCH1      VLPDVKVEFSGANLVVKTVSHRA-PGQTVKVIAALEG--RSLEILDAKINTINDT
SbSPCH2      -LPDVKVEFAGPNLVLKTVSHRS-PGQALKIIAALES--LSLEILHVSVSTVDDT
ZmSPCH2b     RLPDVKVEFAGPNLVLKTVSHRS-PGQALKIIAALES--LPLEILHVSVSTVDDT
ZmSPCH2a     ALPDVKVEFAGPNLVLKTVSHRA-PGQALKIIAALES--LSLQILHVSVSAVDDT
PvSPCH2K     TLPDVKVEFAGPNLVLKTVSRRA-PGQALKIIAALES--LSLEILHVSVSTLDDT
PvSPCH2N     TLPDVKVEFAGPNLVLKTVSRRA-PWQALKIIAALES--LSLEILHVSVSTLDDT
PhSPCH2      ALPDVRVEFAGPNLVLKTVSHRA-PGQALKIIAALES--LSLEILHVSVSTLDDT
SiSPCH2      ALPDVRVEFAGPNLVLKTVSHRA-PGQALKIIAALES--LSLEILHVSVSTVDDT
SvSPCH2      ALPDVRVEFAGPNLVLKTVSHRA-PGQALKIIAALES--LSLEILHVSVSTVDDT
OsSPCH2 (N)  GVPDVRVEFAGPNLVLKTVSHRA-PGQALKIIAALES--LSLEILHVSICTVDDA
OsSPCH2 (K)  GVPDVRVEFAGPNLVLKTVSHRA-PGQALKIIAALES--LSLEILHVSICTVDDA
BdSPCH2      LLPDVKVEFAGPNLVLKTTSHRARPGQVLRIIAALES--LSLEILHVSISTVDDT
```

```
                       |          |      441-473
AtSPCH          MLNSFTIKIGIECQLSAEELAQQIQQTFC----
HvSPCH1         DVNAFTVKIGIECSLSAEELVQEIQQTFS----
TaSPCH1B        DVNAFTVKIGIECSLSAEELVQEIQQTFS----
TaSPCH1A        DVNAFTVKIGIECSLSAEELVQEIQQTFS----
TaSPCH1C        DVNAFTVKIGIECSLSAEELVQEIQQTFS----
BdSPCH1         DVNAFTVKIGIECELSAEELVQEIQQTFS----
OsSPCH1 (N)     AVNSFTVKIGIECELSAEELVQVIQQTFT----
OsSPCH1 (K)     AVNSFTVKIGIECELSAEELVQVIQQTFT----
PvSPCH1K        AVNSFTIKIGIECELSAEELVQEIQQAFS----
PvSPCH1N        AVNSFTIKIGIECELSAEELVQEIQQAFS----
PhSPCH1         AVNSFTIKIGIECELSAEELVQEIQQAFS----
SiSPCH1         AVNSFTIKIGIECELSAEELVQEIQQAFS----
SvSPCH1         AVNSFTIKIGIECELSAEELVQEIQQAFS----
SbSPCH1         AVNSYTIKIGIECELSAEELVQEIQQAFSS---
ZmSPCH1         AVNSYTIKVLLSSS-------------------
SbSPCH2         MVHSFTIKIGIECELSAEELVQEIQQTLL----
ZmSPCH2b        MVHSFTIKIGIECELSAEELVQEIQQTLL----
ZmSPCH2a        MLHSFTIKIGIECELSAEELVQEIQQTLL----
PvSPCH2K        MVHSFTIKVYIDSLFITWCLIVHNNFLIIHVVY
PvSPCH2N        MVHSFTIKIGIECELSAEELVQEIQQTFL----
PhSPCH2         MVHSFTIKIGIECELSAEELVHEIQQTFL----
SiSPCH2         MVHSFTIKIGIECELSAEELVHEIQQTLL----
SvSPCH2         MVHSFTIKIGIECELSAEELVHEIQQTLL----
OsSPCH2 (N)     TVLSFTIKIGIECELSAEELVQEIQQTFL----
OsSPCH2 (K)     TVLSFTIKIGIECELSAEELVQEIQQTFL----
BdSPCH2         MVHSFTIKIGIECELSAEELVQEIRQTFL----
```

Fig. S1 Full amino acid alignment of SPCH1 and SPCH2 proteins from selected plant species. Two *Oryza sativa* L. *japonica* cultivars are included and differentiated by (K) for Kitaake and (N) for Nipponbare, SPCH2 proteins are denoted in lighter grey. Basic-helix-loop-helix (bHLH) is highlighted in green. PEST domain in *Arabidopsis thaliana* annotated with pink highlight and bold text. Putative consensus MAPK phosphorylation sites (P-x-S/T-P) are marked by bold text. SMF (also known as ACT-like) domain is highlighted in yellow. Alignment was performed with CLUSTALW on https://www.genome.jp/tools-bin/clustalw on genes listed in Table S2.

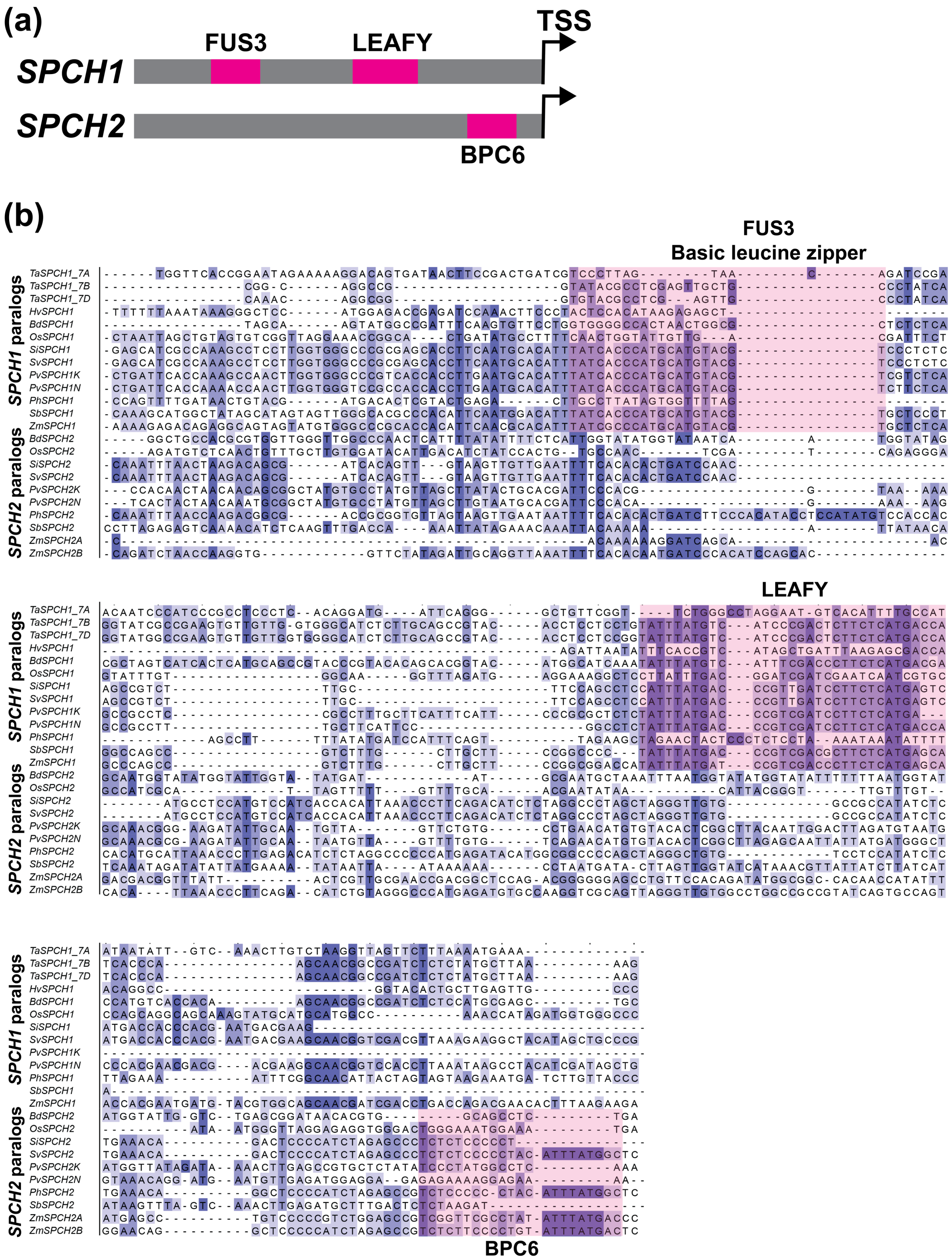

Fig. S2 Predicted paralog-specific transcription factor binding motifs in 5' regulatory sequences. (a) Overview of alignment features and orientation upstream of the transcription start site for *SPCH* paralogs. (b) Section of alignment of 600 bp upstream of the *SPCH* transcription start site for *SPCH* paralogs in *B. distachyon*, *H. vulgare*, *T. aestivum*, *O. sativa*, *P. hallii*, *P. virgatum*, *S. italica*, *S. viridis*, *S. bicolor*, and *Z. mays*. Alignment was performed with Clustal Omega and conserved regions containing TF motifs identified by FIMO with the JASPAR core plant motif dataset are highlighted in pink.

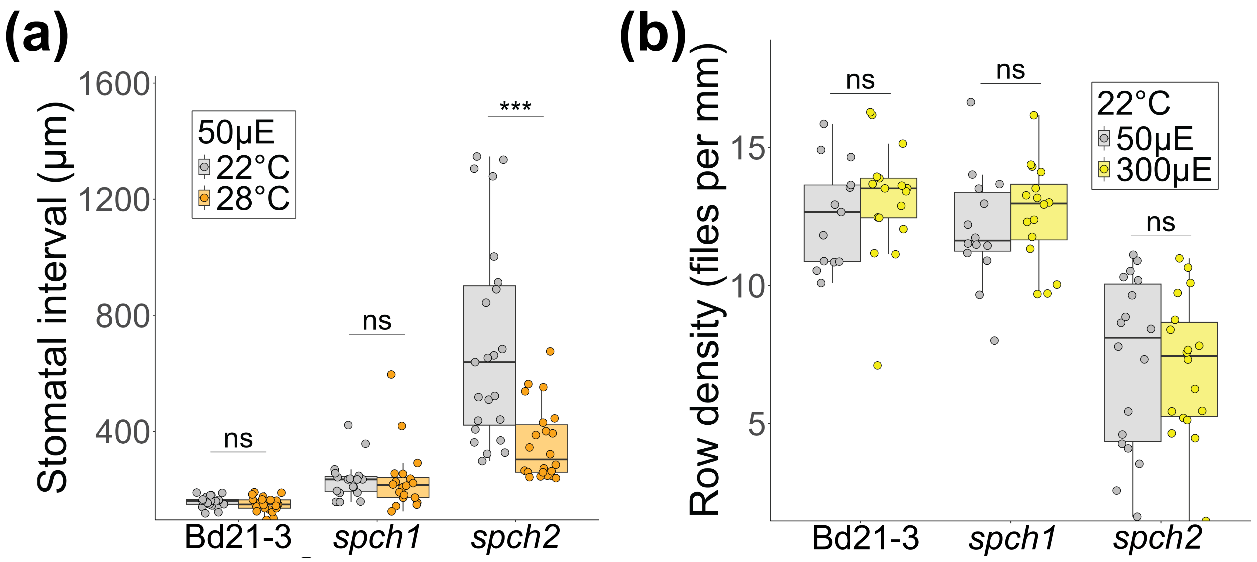

Fig. S3 Row density and stomatal spacing data for additional genotypes and environments. (a) Average space (interval) between two stomata in a row in *B. distachyon* plants grown at 22°C or 28°C, 50µE conditions. Stomata spacing does not decrease with temperature except in *bdspch2* plants. (b) Row density of stomata cell files in plants grown at 50µE or 300µE, 22°C conditions. Row density does not increase with light intensity. Dots represent regions across multiple leaves (n > 15 per genotype and condition). Boxes in panels (a) and (b) indicate the median (horizontal line) and the interquartile range (box: 25th–75th percentiles). Outliers are plotted as individual points. Two-way ANOVA with Tukey post-hoc reporting the effect of condition while controlling for leaf identity of each region (*** < 1e-3).

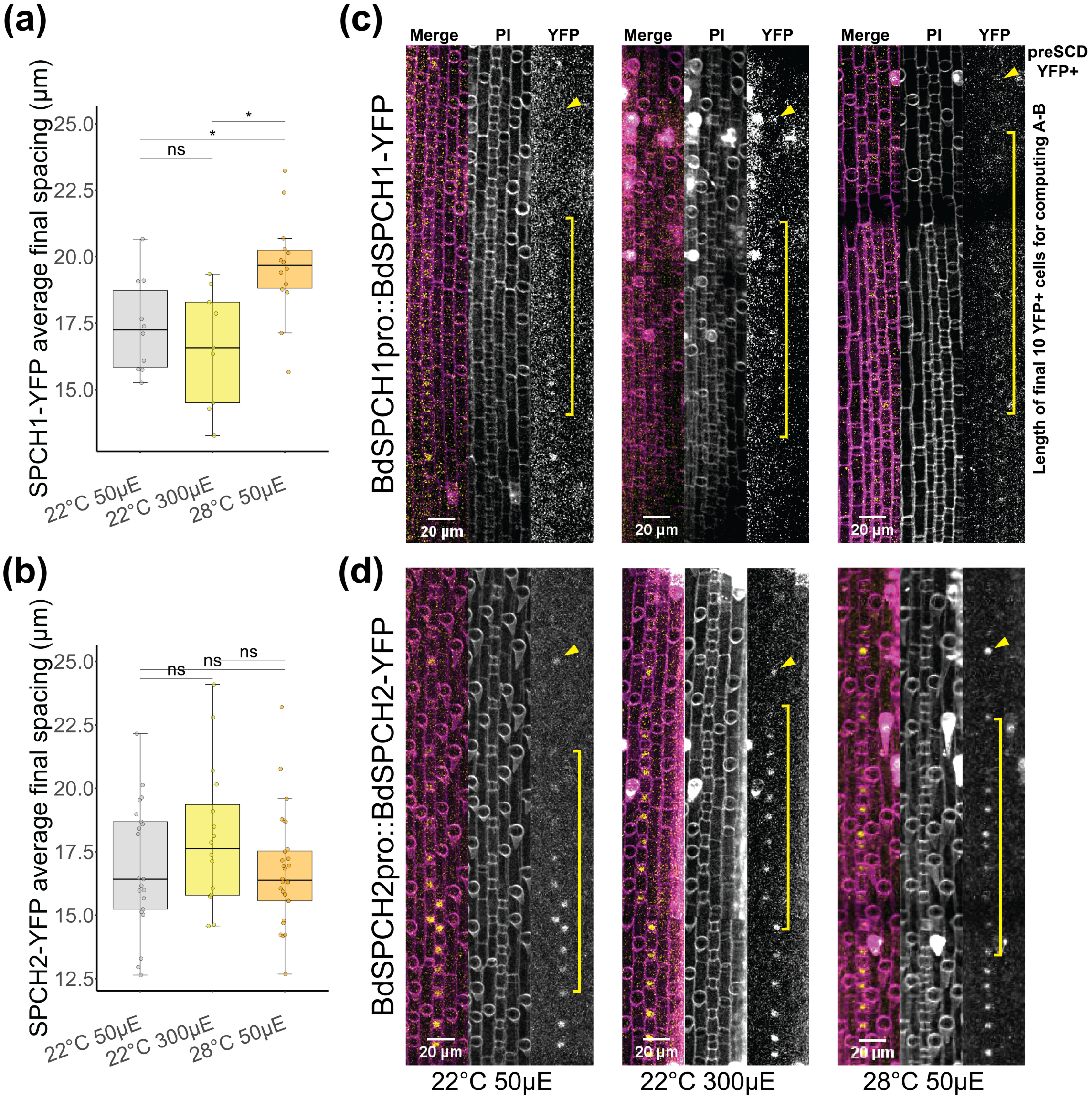

Fig. S4 Protein accumulation of BdSPCH paralogs extends along the developmental zone in a paralog-by-condition specific manner. Average space between the ten YFP-positive cells before the final pre-SCD cell in (a) BdSPCH1:BdSPCH1-YFP or (b) BdSPCH2:BdSPCH2-YFP. Dots represent cell file averages across multiple *B. distachyon* leaves (n > 8 per genotype and condition). Boxes in panels (a) and (b) indicate the median (horizontal line) and the interquartile range (box: 25th–75th percentiles). Two-way ANOVA with Tukey post-hoc reporting the effect of condition while controlling for leaf identity of each cell file (* < 0.05). (c,d) Representative epidermal micrographs illustrating distribution of YFP fluorescence in each cell file before final pre-SCD expression. Yellow brackets indicate examples of 10 measured cells and yellow arrowheads indicate pre-SCD cell.

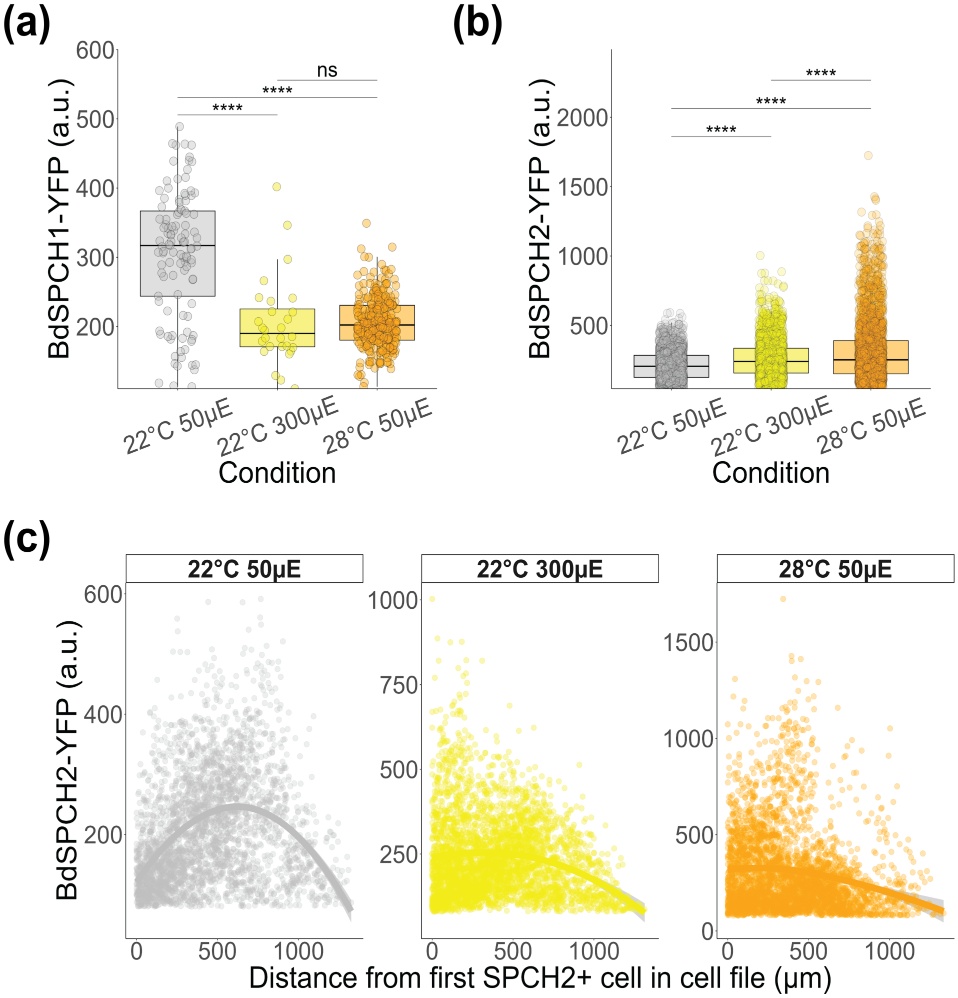

Fig. S5 SPCH reporter per cell intensity is insufficient to explain paralog-specific environmental response. Intensity of reporter fluorescence on a per cell basis under multiple conditions from (a) BdSPCH1:BdSPCH1-YFP or (b) BdSPCH2:BdSPCH2-YFP Two-way ANOVA with Tukey post-hoc (**** < 1-e4) reporting the effect of condition while controlling for *B. distachyon* leaf identity (n > 8 per genotype and condition) of each cell (n > 100). Boxes in panels (a) and (b) indicate the median (horizontal line) and the interquartile range (box: 25th–75th percentiles). (c) Cell fluorescence intensity of BdSPCH2:BdSPCH2-YFP by distance from the first fluorescent cell within that cell file summed across all cell files for each condition. Smoothed conditional means line (LOESS regression) shows shared trend across conditions of increasing and then decreasing fluorescence intensity.

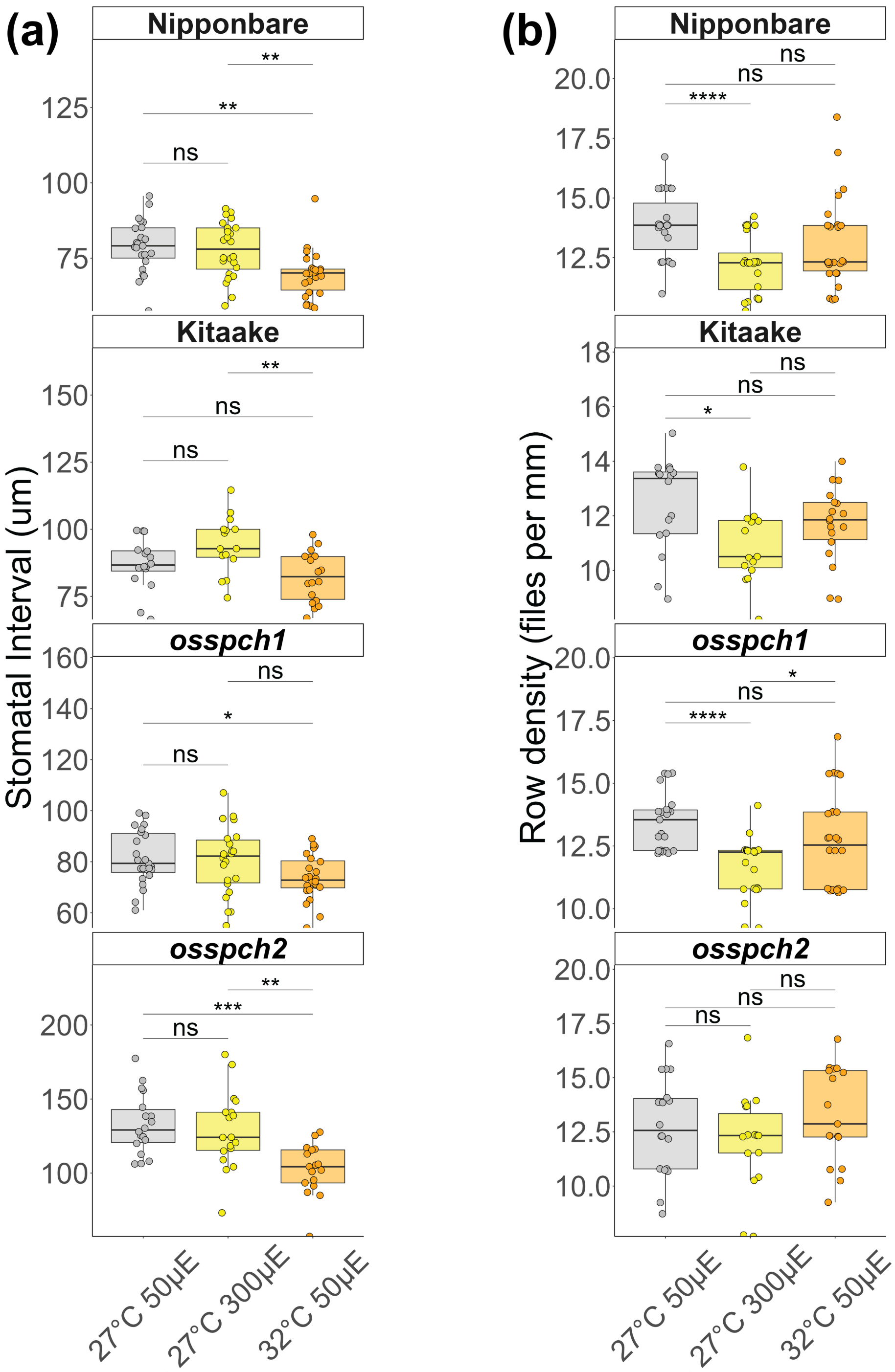

Fig. S6 *O. sativa* stomatal density response driven by alternative mechanisms than those used by B. *distachyon*. (a) Average space (interval) between two stomata in a row across *O.sativa* genotypes and conditions. (b) Row density of stomata cell files across *O.sativa* genotypes and conditions. Dots represent regions across multiple leaves (Kitaake n > 7, others n > 16 per condition). Boxes in panels (a) and (b) indicate the median (horizontal line) and the interquartile range (box: 25th–75th percentiles). Two-way ANOVA with Tukey post-hoc reporting the effect of condition while controlling for leaf identity of each region (* < 0.05, ** < 0.01, *** < 1e-3, **** < 1e-4).

Table S1 Primer sequences used for genotyping and expression analysis

**Primer Name**	**Primer Sequence**	**Purpose**
bdspch1-F	GCACGTACACCACTGATCAT	*bdspch1* genotyping
bdspch1-R	ACATAGAAGCACGGCATGAG	*bdspch1* genotyping
bdspch2-F	ACACATGCACAGAACGACCT	*bdspch2* genotyping
bdspch2-R	AACTCCACCTTCACGTCAGG	*bdspch2* genotyping
osspch1-F	CGACCTCTTCACCATCCTCG	*osspch1-1-c* genotyping
osspch1-R	GGCGAGGTTCTCGTTCATCT	*osspch1-1-c* genotyping
osspch2-F	AACGGTCTACTCTCTCTCTCTC	*osspch2-t* genotyping
osspch2-R	GCACTGTAGTACTCTTGGTCAC	*osspch2-t* genotyping
BdSPCH1qpcr-F	CACCGTCAAGATTGGAATCGAGTG	qPCR primer for *BdSPCH1*
BdSPCH1qpcr-R	TCACGAGAACGTTTGCTGAATCTC	qPCR primer for *BdSPCH1*
BdSPCH2qpcr-F	TAGCACCGTCGACGACACTATG	qPCR primer for *BdSPCH2*
BdSPCH2qpcr-F	TTCGGCGCTAAGCTCACATTCG	qPCR primer for *BdSPCH2*
BdUBC18qpcr-F	GTCACCCGCAATGACTGTAAGTTC	qPCR primer for *BdUBC18*
BdUBC18qpcr-R	TTGTCTTGCGGACGTTGCTTTG	qPCR primer for *BdUBC18*
AtUBC18qpcr-F	CAGTCTGTGTGTAGAGCTATCA	qPCR primer for *AtUBC18*
AtUBC18qpcr-R	ATCTTAGAAGATTCCCTGAGTC	qPCR primer for *AtUBC18*

Table S2 Data sources for *SPEECHLESS (SPCH)* paralog sequences used in this paper

**Species**	**Gene label**	**Gene Id**	**Genome version (accessed from phytozome.org)**
Brachypodium distachyon	SPCH1	Bradi1g38650	v3.2
Brachypodium distachyon	SPCH2	Bradi3g09670	v3.2
Hordeum vulgare	SPCH1	HORVU7Hr1G079250	r1
Oryza sativa Nipponbare	SPCH1	LOC_Os06g33450	v7
Oryza sativa Nipponbare	SPCH2	LOC_Os02g15760	v7
Oryza sativa Kitaake	SPCH1	OsKitaake06g172500	v3.1
Oryza sativa Kitaake	SPCH2	OsKitaake02g112600	v3.1
Panicum hallii	SPCH1	Pahal.4G131400	v3.2
Panicum hallii	SPCH2	Pahal.1G118900	v3.2
Panicum virgatum	SPCH1K	Pavir.4KG254300	v5.1
Panicum virgatum	SPCH1N	Pavir.4NG174200	v5.1
Panicum virgatum	SPCH2K	Pavir.1KG252700	v5.1
Panicum virgatum	SPCH2N	Pavir.1NG142600	v5.1
Setaria italica	SPCH1	Seita.4G167700	v2.2
Setaria italica	SPCH2	Seita.1G013200	v2.2
Setaria viridis	SPCH1	Sevir.4G135400	v4.1
Setaria viridis	SPCH2	Sevir.1G013100	v4.1
Sorghum bicolor	SPCH1	Sobic.010G152700	v5.1
Sorghum bicolor	SPCH2	Sobic.004G115000	v5.1
Triticum aestivum	SPCH1A	TraesCS7A03G0857600	ChineseSpring v2.1

Triticum aestivum	SPCH1B	TraesCS7B03G0653200	ChineseSpring v2.1
Triticum aestivum	SPCH1C	TraesCS7D03G0780300	ChineseSpring v2.1
Zea mays	SPCH1	Zm00001d046096	RefGenv4
Zea mays	SPCH2a	Zm00001d016095	RefGenv4
Zea mays	SPCH2b	Zm00001d053391	RefGenv4
Arabidopsis thaliana	SPCH	AT5G53210	Araport11
