# Supplementary material for: SPEECHLESS duplication in grasses expands potential for environmental regulation of stomatal development
Source: bioRxiv. 2025 Jul 30:2025.07.29.667563. Preprint. [Version 1] doi: 10.1101/2025.07.29.667563 (PMC12327695; doi:10.1101/2025.07.29.667563)
Supplement: Supplementary file 1 [file media-1.pdf]

Supplemental Data:

SPEECHLESS duplication in grasses expands potential for environmental regulation of stomatal development

Joel M. Erberich[1], Britney Bennett[1], Dominique C. Bergmann[1,2]*

Supplemental Figures 1-5

Supplemental Tables 1-2

1

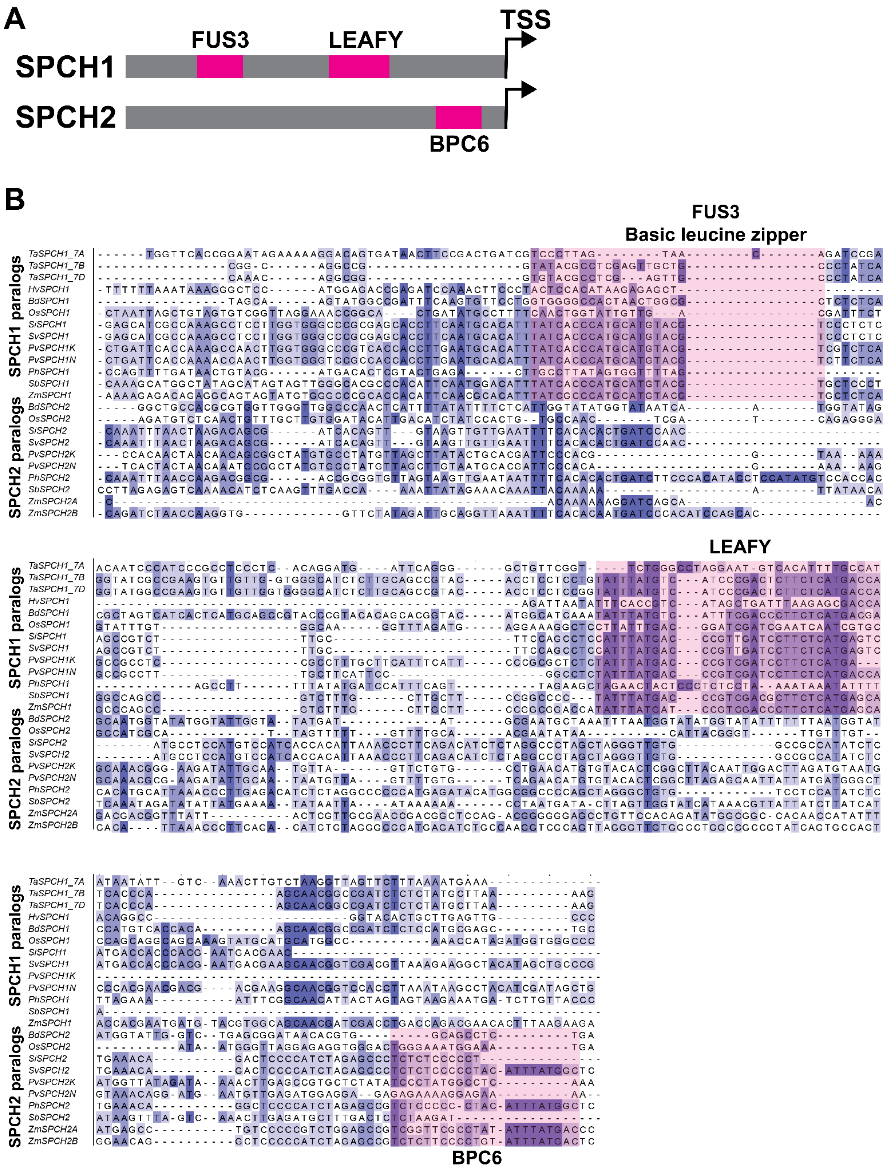

Supplemental Figure 1 (related to Fig. 1) **Predicted paralog specific motifs.** A) Overview of alignment features and orientation ~100 bp upstream from the transcription start site for SPCH paralogs. B) Section of alignment of 600 bp upstream from SPCH transcription start site for SPCH paralogs in *B. distachyon*, *H. vulgare*, *T. aestivum*, *O. sativa*, *P. hallii*, *P. virgatum*, *S. italica*, *S. viridis*, *S. bicolor*, and *Z. mays*. Alignment was performed with Clustal Omega and conserved regions containing TF motifs identified by FIMO with the JASPAR core plant motif dataset are highlighted in pink.

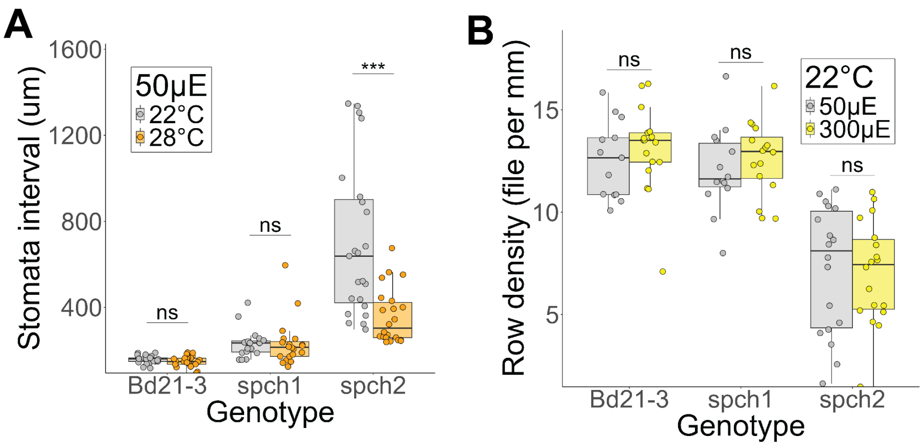

Supplemental Figure 2 (related to Fig. 2) **Row density and stomatal spacing data for additional genotypes and environments.** A) Average space (interval) between two stomata in a row in plants grown at 22°C or 28°C, 50μE conditions. Stomata spacing does not decrease with temperature except in *bdspch2* plants. B) Row density of stomata cell files in plants grown at 50μE or 300μE, 22°C conditions. Row density does not increase with light intensity. Dots represent regions across multiple leaves (n > 15 per genotype and condition). Two-way ANOVA with Tukey post-hoc reporting the effect of condition while controlling for leaf identity of each region (*** < 1e-3).

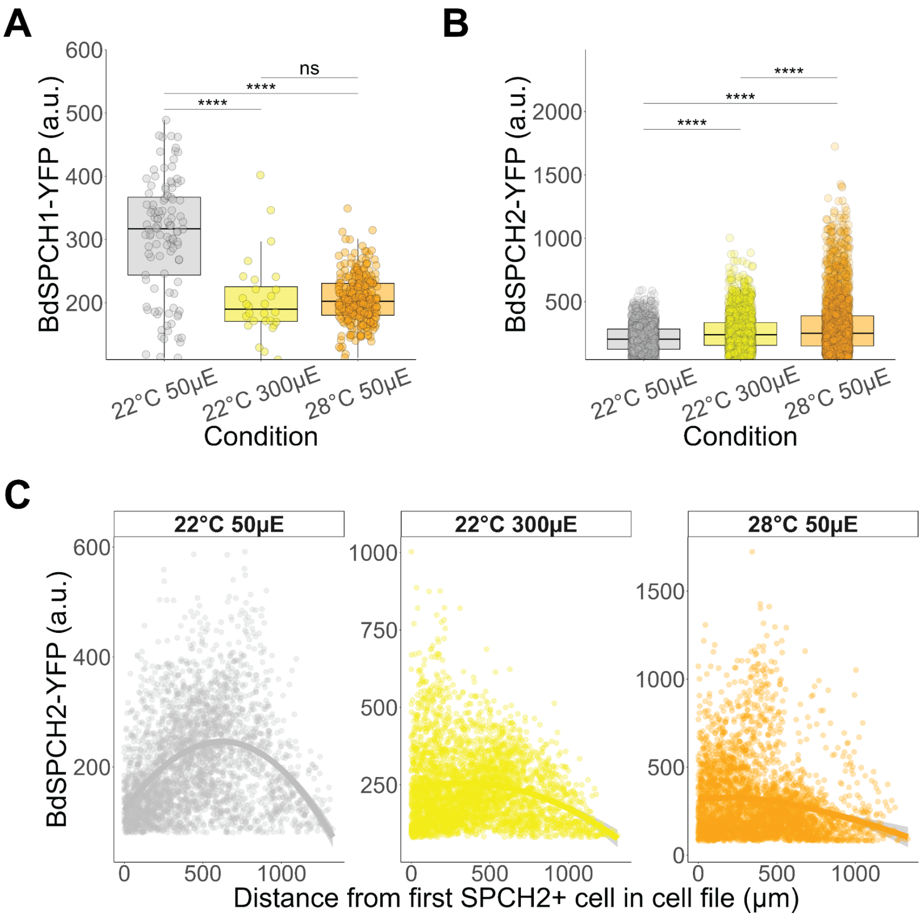

Supplemental Figure 3 (related to Fig. 3) **SPCH reporter per cell intensity is insufficient to explain paralog-specific environmental response.** Intensity of reporter fluorescence on a per cell basis under multiple conditions from A) BdSPCH1:BdSPCH1-YFP or B) BdSPCH2:BdSPCH2-YFP Two-way ANOVA with Tukey post-hoc (**** < 1-e4) reporting the effect of condition while controlling for leaf identity (n > 8 per genotype and condition) of each cell (n > 100). C) Cell fluorescence intensity of BdSPCH2:BdSPCH2-YFP by distance from the first fluorescent cell within that cell file summed across all cell files for each condition. Smoothed conditional means line (LOESS regression) shows shared trend across conditions of increasing and then decreasing fluorescence intensity.

4

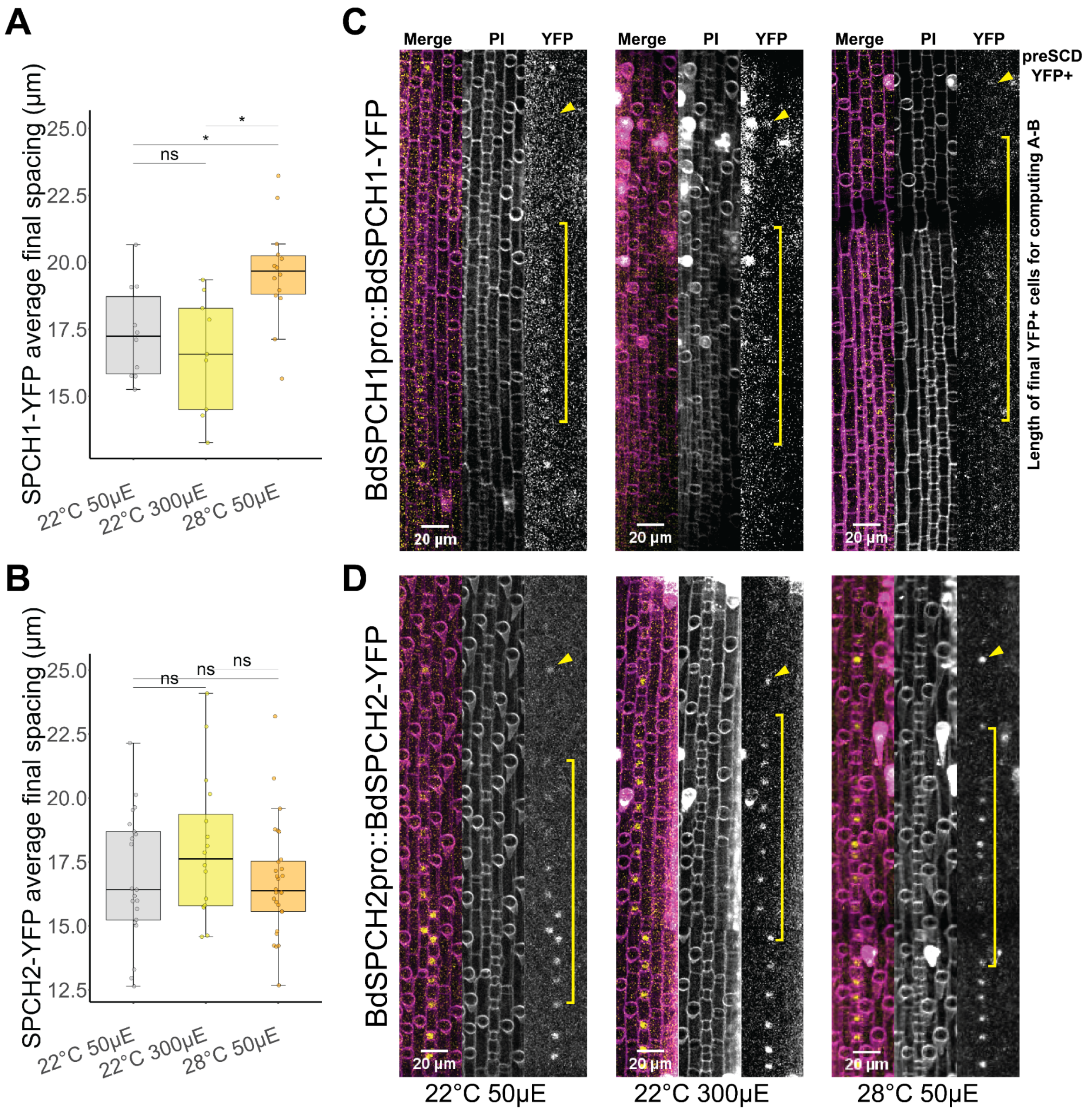

Supplemental Figure 4 (related to Fig. 3) **Protein accumulation of BdSPCH paralogs extends along the developmental zone in a paralog-by-condition specific manner.** Average space between the ten YFP-positive cells before the final pre-SCD cell in (A) BdSPCH1:BdSPCH1-YFP or (B) BdSPCH2:BdSPCH2-YFP. Dots represent cell file averages across multiple leaves (n > 8 per genotype and condition). Two-way ANOVA with Tukey post-hoc reporting the effect of condition while controlling for leaf identity of each cell file (* < 0.05). C-D) Representative epidermal micrographs illustrating distribution of YFP fluorescence in each cell file before final pre-SCD expression. Yellow bracket indicate examples of 10 measured cells and yellow arrowhead indicates pre-SCD cell.

5

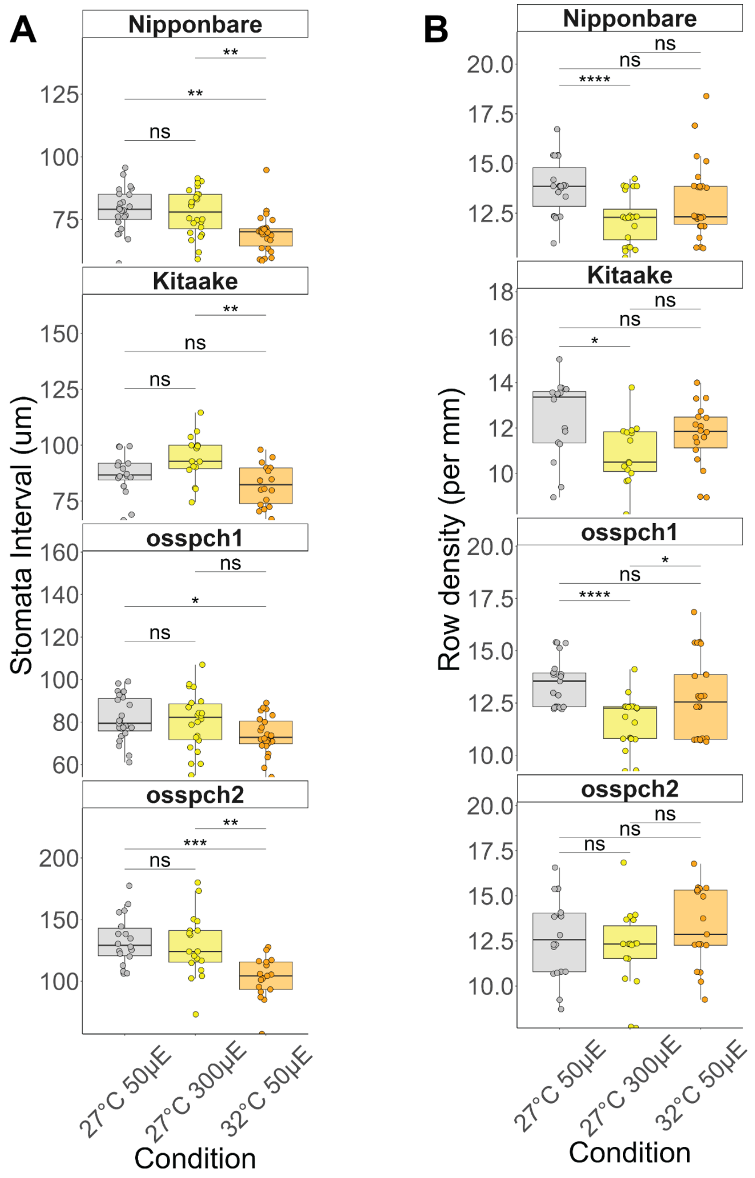

Supplemental Figure 5 (related to Fig. 4) **Rice stomata density response driven by alternative mechanisms than *B. distachyon*.** A) Average space (interval) between two stomata in a row across *O.sativa* genotypes and conditions. B) Row density of stomata cell files across *O.sativa* genotypes and conditions. Dots represent regions across multiple leaves (Kitaake n > 7, others n > 16 per condition). Two-way ANOVA with Tukey post-hoc reporting the effect of condition while controlling for leaf identity of each region (* < 0.05, ** < 0.01, *** < 1e-3, **** < 1e-4).

6

Table S1: Primer sequences

Primer Name	Primer Sequence	Purpose
bdspch1-F	GCACGTACACCACTGATCAT	bdspch1 genotyping
bdspch1-R	ACATAGAAGCACGGCATGAG	bdspch1 genotyping
bdspch2-F	ACACATGCACAGAACGACCT	bdspch2 genotyping
bdspch2-R	AACTCCACCTTCACGTCAGG	bdspch2 genotyping
osspch1-F	CGACCTCTTCACCATCCTCG	osspch1-1-c genotyping
osspch1-R	GGCGAGGTTCTCGTTCATCT	osspch1-1-c genotyping
osspch2-F	AACGGTCTACTCTCTCTCTCTC	osspch2-t genotyping
osspch2-R	GCACTGTAGTACTCTTGGTCAC	osspch2-t genotyping
BdSPCH1qpcr-F	CACCGTCAAGATTGGAATCGAGTG	qPCR primer for BdSPCH1
BdSPCH1qpcr-R	TCACGAGAACGTTTGCTGAATCTC	qPCR primer for BdSPCH1
BdSPCH2qpcr-F	TAGCACCGTCGACGACACTATG	qPCR primer for BdSPCH2
BdSPCH2qpcr-F	TTCGGCGCTAAGCTCACATTCG	qPCR primer for BdSPCH2
BdUBC18qpcr-F	GTCACCCGCAATGACTGTAAGTTC	qPCR primer for BdUBC18
BdUBC18qpcr-R	TTGTCTTGCGGACGTTGCTTTG	qPCR primer for BdUBC18
AtUBC18qpcr-F	CAGTCTGTGTGTAGAGCTATCA	qPCR primer for AtUBC18
AtUBC18qpcr-R	ATCTTAGAAGATTCCCTGAGTC	qPCR primer for AtUBC18

7

Table S2: Species and SPEECHLESS paralogs

Species	Gene label	Gene Id	Genome version
Brachypodium distachyon	SPCH1	Bradi1g38650	v3.2
Brachypodium distachyon	SPCH2	Bradi3g09670	v3.2
Hordeum vulgare	SPCH1	HORVU7Hr1G079250	r1
Oryza sativa	SPCH1	LOC_Os06g33450	v7
Oryza sativa	SPCH2	LOC_Os02g15760	v7
Pancum hallii	SPCH1	Pahal.4G131400	v3.2
Pancum hallii	SPCH2	Pahal.1G118900	v3.2
Pancum virgatum	SPCH1K	Pavir.4KG254300	v5.1
Pancum virgatum	SPCH1N	Pavir.4NG174200	v5.1
Pancum virgatum	SPCH2K	Pavir.1KG252700	v5.1
Pancum virgatum	SPCH2N	Pavir.1NG142600	v5.1
Setaria italica	SPCH1	Seita.4G167700	v2.2
Setaria italica	SPCH2	Seita.1G013200	v2.2
Setaria viridis	SPCH1	Sevir.4G135400	v4.1
Setaria viridis	SPCH2	Sevir.1G013100	v4.1
Sorghum bicolor	SPCH1	Sobic.010G152700	v5.1
Sorghum bicolor	SPCH2	Sobic.004G115000	v5.1
Triticum aestivum	SPCH1A	TraesCS7A03G0857600	ChineseSpring v2.1
Triticum aestivum	SPCH1B	TraesCS7B03G0653200	ChineseSpring v2.1
Triticum aestivum	SPCH1C	TraesCS7D03G0780300	ChineseSpring v2.1
Zea mays	SPCH1	Zm00001d046096	RefGenv4
Zea mays	SPCH2a	Zm00001d016095	RefGenv4
Zea mays	SPCH2b	Zm00001d053391	RefGenv4

 8
